# Marrubiin Inhibits Peritoneal Inflammatory Response Induced by Carrageenan Application in C57 Mice

**DOI:** 10.3390/ijms25084496

**Published:** 2024-04-19

**Authors:** Niko S. Radulović, Miljana R. Đorđević Zlatković, Nikola M. Stojanović, Milan S. Nešić, Dragan B. Zlatković, Milena S. Potić Floranović, Dragana S. Tričković Vukić, Pavle J. Randjelovic

**Affiliations:** 1 Department of Chemistry, Faculty of Sciences and Mathematics, University of Niš, 18000 Niš, Serbia; miljana.djordjevic@pmf.edu.rs (M.R.Đ.Z.); nesicmilanvl@gmail.com (M.S.N.); dragan.zlatkovic@gmail.com (D.B.Z.); 2 Department of Physiology, Faculty of Medicine, University of Niš, 18000 Niš, Serbia; nikola.st90@yahoo.com (N.M.S.); pavleus@gmail.com (P.J.R.); 3 Scientific Research Centre for Biomedicine, Faculty of Medicine, University of Niš, 18000 Niš, Serbia; milenapotic@yahoo.com (M.S.P.F.); draganavukic18@gmail.com (D.S.T.V.)

**Keywords:** marrubiin, peritoneal inflammation, inflammatory infiltrate, scanning electron microscopy

## Abstract

Marrubiin is a diterpene with a long history of a wide range of biological activities. In this study, the anti-inflammatory effects of marrubiin were investigated using several in vitro and in vivo assays. Marrubiin inhibited carrageenan-induced peritoneal inflammation by preventing inflammatory cell infiltration and peritoneal mast cell degranulation. The anti-inflammatory activity was further demonstrated by monitoring a set of biochemical parameters, showing that the peritoneal fluid of animals treated with marrubiin had lower levels of proteins and lower myeloperoxidase activity compared with the fluid of animals that were not treated. Marrubiin exerted the most pronounced cytotoxic activity towards peripheral mononuclear cells, being the main contributors to peritoneal inflammation. Additionally, a moderate lipoxygenase inhibition activity of marrubiin was observed.

## 1. Introduction

**Figure 1:**
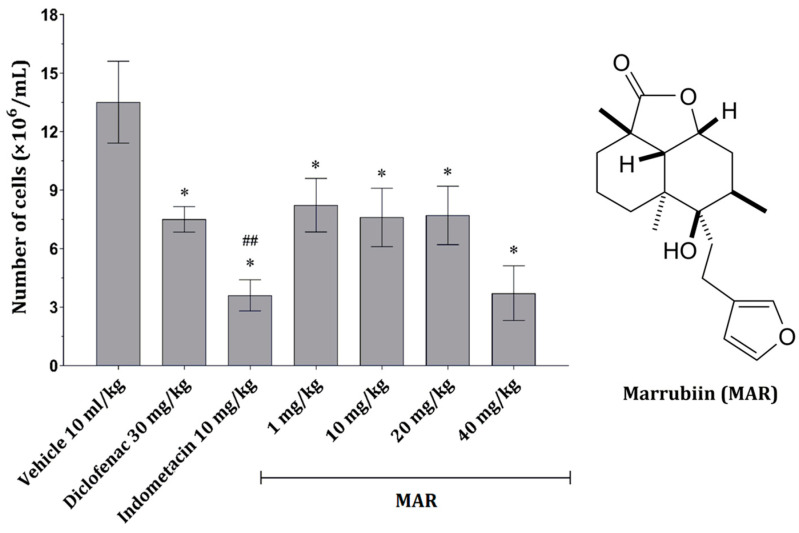
(**Left**) The number of peritoneal inflammatory cells obtained from mice belonging to different experimental groups; data given as mean ± SD (*n* = 6), compared using one-way ANOVA followed by Tukey’s post hoc test, * *p* < 0.001 vs. vehicle-treated animals; ^##^ *p* < 0.01 vs. diclofenac-treated animals. (**Right**) Chemical structure of marrubiin (MAR).

Marrubiin (MAR, Figure 1) is a furanic labdane diterpene that can be found in the aerial parts of several *Marrubium* and *Leonotis* plant species of the family Lamiaceae [1]. The compound was initially isolated from *Marrubium vulgare* and described in the 1850s. However, the exact structure of marrubiin eluded researchers for over a century [2] until its correct connectivity and stereochemistry were determined in the 1960s [3]. Subsequent studies revealed that MAR was not restricted to white horehound (*M. vulgare*) but is characteristic of the whole genus [2]. MAR, as well as marrubiin-containing extracts, has been extensively studied in various biological assays, revealing antioxidant and antigenotoxic [4], antioedematogenic [5], analgesic [6], antinociceptive [7], and antidiabetic [8] properties. For an extensive review of the (ethno)pharmacological studies on MAR, the reader is referred to the review article by Popoola et al. [1]. MAR shows a positive druglikeness with the five most common filters (Lipinski, Ghose, Veber, Egan, and Muegge [9]).

Inflammation is a natural process that occurs when the body is exposed to harmful stimuli, such as pathogens, toxins, or physical injury. While acute inflammation is a necessary and beneficial response to these stimuli, chronic inflammation can contribute to the development and progression of many diseases, including cancer, cardiovascular disease, and autoimmune disorders. Anti-inflammatory testing allows us to identify compounds that can reduce inflammation and potentially be used as therapeutic agents for treating or preventing inflammatory diseases. Anti-inflammatory testing is typically performed using in vitro and in vivo assays, which can evaluate the effects of a substance on various aspects of the inflammatory response, such as the production of proinflammatory cytokines, the activation of immune cells, and the expression of inflammatory mediators.

The aim of this study was to investigate the anti-inflammatory effect of MAR through various in vitro and in vivo assays, including the carrageenan-induced peritonitis test and inflammatory-cell viability assay. Additionally, lipoxygenase inhibition activity was also examined.

## 2. Results

### 2.1. Isolation of MAR

MAR was isolated from *Marrubium peregrinum* L. (Lamiaceae) extract (hexane/Et_2_O/ethyl acetate/MeOH) via silica gel and Sephadex chromatography as a white powder. The structure was initially determined via NMR analysis (1D and 2D) and unequivocally confirmed through comparing ^1^H and ^13^C NMR chemical shifts (in CDCl_3_) with those reported in the literature [10]. Graphical ^1^H and ^13^C spectra are given in the Supplementary Materials (Figures S1–S3).

### 2.2. In Vivo Anti-Inflammatory Activity

#### 2.2.1. MAR Dose-Dependently Decreases the Number of Peritoneal Inflammatory Cells

A significant effect on cell numbers could be seen in the treatment with MAR applied in all four doses. However, the effect was most pronounced at a dose of 40 mg/kg (Figure 1). Compared with the positive control group (carrageenan-treated animals), a significant decrease in inflammatory cells was observed in animals treated with indometacin (10 mg/kg). In contrast, the effect of diclofenac (30 mg/kg) only slightly influenced the number of cells in the peritoneal exudate (Figure 1).

#### 2.2.2. Scanning Electron Microscopy (SEM)

**Figure 2:**
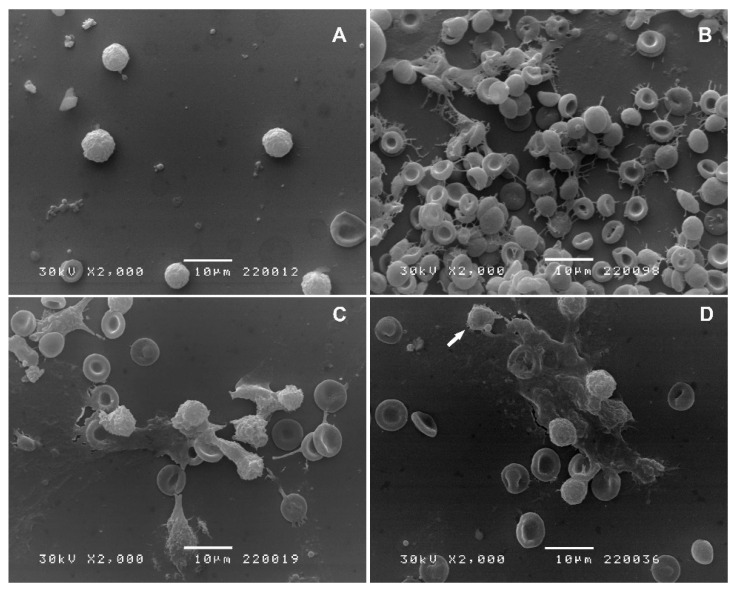
Scanning electron microscopic images of cells from the peritoneal cavity of mice belonging to the control (**A**), carrageenan (**B**), indometacin 10 mg/kg + carrageenan (**C**), and MAR 40 mg/kg + carrageenan (**D**) groups. (**A**) Standard sized and round white blood cells (WBCs); (**B**) massive WBC infiltration with numerous apoptotic bodies, cell protrusions, as well as fibers with entrapped erythrocytes; (**C**) decreased number of WBCs and occasional cells with degenerative changes; (**D**) reduced number of WBCs with degenerative changes and apoptosis (arrow).

In the control group of animals, white blood cells (WBCs) were small, with a size equivalent to the size of the erythrocyte. Even though cell adhesion was noted, the microvilli of these cells were short, scarce, and unevenly distributed (Figure 2A). Carrageenan stimulation induced an inflammatory response (Figure 2B). Massive erythrocyte aggregation was caused by extravasation and endothelial damage. Fibrillar structures may represent neutrophil extracellular traps (NETs). Indometacin reduced the inflammatory response (Figure 2C) by decreasing erythrocyte aggregation and formation of inflammatory debris. MAR administration prior to carrageenan stimulation decreased the extent of inflammatory response and caused occasional apoptosis (Figure 2D; arrow).

#### 2.2.3. MAR Dose-Dependently Reduces the Inflammatory Parameters in Exudate Fluid

Protein concentrations were statistically significantly lower in all experimental groups receiving MAR and those receiving the anti-inflammatory drug than in the vehicle-treated group (Table 1). The same pattern of activity was observed for the activity of myeloperoxidase (MPO) in the peritoneal fluid, i.e., a significant decrease in groups treated with either MAR or anti-inflammatory drugs compared with the vehicle-treated animals (Table 1). Levels of reduced glutathione (GSH) in the peritoneal fluid were not found to be different among different groups of animals (Table 1). The glutathione peroxidase (GPx) activity was significantly decreased in animals treated with the vehicle and carrageenan. In contrast, the activity of this enzyme remained higher in those treated with MAR in all tested doses or diclofenac (Table 1). Interestingly, in animals treated with indometacin and carrageenan, the activity was even lower than in the animals treated with the vehicle and carrageenan (Table 1).

#### 2.2.4. MAR Prevents Mast Cell Degranulation in the Mesenterial Tissue of Animals Treated with Carrageenan

**Figure 3:**
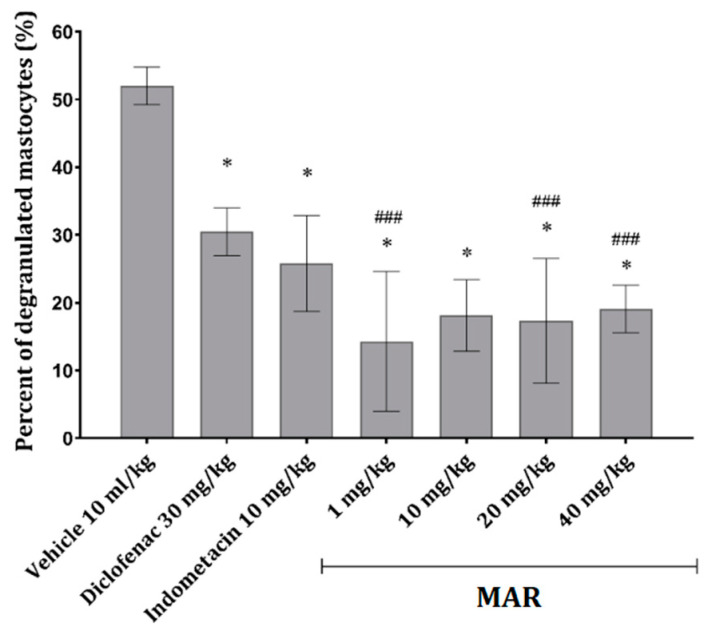
Percent of degranulated mastocytes in mesenterial tissue obtained from mice belonging to different experimental groups; data given as mean ± SD (*n* = 6), compared using one-way ANOVA followed by Tukey’s post hoc test, * *p* < 0.001 vs. vehicle-treated animals, ^###^ *p* < 0.05 vs. diclofenac-treated animals.

Compared with the control (carrageenan only), where a significant increase in degranulated mast cells was observed, a significant decrease in degranulation could be observed in animals treated with MAR in all four doses (Figure 3). Interestingly, the most pronounced effect was visible in animals treated with MAR at 1 mg/kg. The effects of diclofenac (30 mg/kg) and indometacin (10 mg/kg) were noticeable and uniform; however, their effect was significantly less pronounced than that of MAR (Figure 3).

### 2.3. Time Lapse Study—Effect of MAR on Inflammatory-Cell Viability

MAR application in concentrations ranging from 10^−8^ to 10^−4^ M exerted modest cytotoxic potential towards granulocytes, peripheral mononuclear cells (PMNCs), and spleen lymphocytes (Table 2). The most pronounced effect was observed in a short culture of PMNCs, in which, after 3 h, a statistically significant decrease in the number of viable cells (the ones able to metabolize MTT) was noted (Table 2). The second most sensitive cells to the impact of MAR were granulocytes, and the most pronounced cytotoxicity towards these cells was seen after 5 h of incubation (Table 2). The most resistant cell type towards the action of MAR was spleen lymphocyte culture, in which after 5 h of incubation, only the highest tested concentration of MAR exerted a statistically significant decrease in cell viability (Table 2).

## 3. Discussion

Carrageenan is a commonly used inflammatory stimulus in preclinical experimental models for evaluating the anti-inflammatory properties of various compounds. It induces neutrophil migration and activation, along with the activation of peritoneal macrophages to produce proinflammatory cytokines such as TNF-α and IL-1β. These cytokines, through the activation of tyrosine kinase signaling pathways, promote the production of reactive oxygen species (ROS) [11]. TNF-α, via ROS production, mediates endothelial destruction, potentially leading to the presence of inflammatory cells and erythrocytes in the peritoneal space [12]. Indometacin and diclofenac, common positive control drugs, exert their anti-inflammatory effects by inhibiting prostaglandin synthesis, suppressing cyclooxygenase (COX), and modulating K^+^ channel opening, which are crucial mediators of the inflammatory response. Moreover, these drugs often induce inflammatory cell apoptosis and inhibit neutrophil aggregation, partially mediated through prostaglandin signaling [13]. Consequently, a decrease in the number of inflammatory cells in the peritoneal cavity was observed in mice treated with carrageenan alone or in combination with diclofenac/indometacin (Figure 1).

Methanolic extract of *M. vulgare* has demonstrated cytotoxic effects on various cancer cell lines under in vitro conditions [14]. Additionally, studies have shown that the extract induces apoptosis and inhibits proliferation in malignant cells, involving the activation of caspases and modulation of pro- and anti-apoptotic genes [15]. The findings of the present study help elucidate the observed activity of *M. vulgare* extract [14,15], pointing to the fact that MAR might be one of the main activity carriers since it led to the reduction of inflammatory cell numbers in the peritoneal cavity following carrageenan exposure (Figure 1).

Scanning electron microscopy (SEM) images from the carrageenan group suggest the formation of neutrophil extracellular traps (NETs), associated with carrageenan-induced inflammation [16]. These structures, comprising degraded neutrophil components and chromatin, are released as a result of neutrophil membrane disintegration, a process termed NETosis. Additionally, the observed fibrinous trap-like structures could be interpreted as cytonemes, tubulovesicular extensions produced by stimulated neutrophils, which play roles in antibacterial activity and cell communication [17]. In animals treated with MAR before carrageenan exposure for the first time, we observed a decrease in the presence of NETs, as well as a significant reduction in inflammatory and red blood cells (Figure 2D). Additionally, occasional apoptosis observed during SEM analysis of samples from MAR-treated animals further confirms the pro-apoptotic potential of *M. vulgare* extract [14]. Thus, a decrease in different blood cell types in the peritoneal fluid of treated animals, along with altered cell morphology, further supports the impact of *M. vulgare* extract on inflammatory processes (Figure 2D). Even more pronounced results were observed in peritoneal fluid cells obtained from animals treated with indometacin and carrageenan (Figure 2C), confirming the efficacy of this standard drug [13].

The anti-inflammatory effects of the *M. vulgare* extract, including the reduction in microvascular leakage and edema by various stimuli, have been previously assessed [5]. In the current study, MAR demonstrated systemic inhibition of microvascular leakage and ear edema induced by carrageenan, histamine, and substance P. This inhibition of microvascular permeability is likely to have contributed to the observed reduction in WBC and erythrocyte extravasation in the inflammatory model. Moreover, MAR showed potential anti-edematous effects, evidenced by reduced fluid accumulation in the abdomen of animals exposed to both carrageenan and MAR (Table 1). Protein extravasation, indicative of capillary permeability or damage during inflammation, was also diminished in animals treated with the extract [18,19].

Mast cell degranulation, a key event in allergic reactions, is triggered by histamine and complement molecules, leading to the release of proinflammatory mediators. Both standard drugs and MAR extract decreased mast cell degranulation in animals with carrageenan-induced peritonitis (Figure 3), potentially mitigating vasodilation and extravasation [11].

Myeloperoxidase (MPO), an enzyme derived from inflammatory cells, plays a significant role in inflammation by generating reactive molecules that can damage surrounding tissue [20]. The decrease in inflammatory cell numbers observed in animals treated with MAR or standard drugs may explain the reduction in free MPO activity (Figure 1 and Figure 2C,D; Table 1). MAR may also directly inhibit MPO activity, albeit to a lesser extent than indometacin [21].

MAR, the major diterpenoid in the *M. vulgare* extract, exhibits potent antioxidant activity in vitro [22,23]. This activity is likely to interfere with ROS formation, which is implicated in inflammation-induced oxidative stress. Increased ROS leads to the consumption of antioxidants in the peritoneal fluid, including among them GSH [24] and GPx [25], which were evaluated here (Table 1). The effect of MAR on peritoneal fluid glutathione peroxidase (GPx) levels further supports its antioxidative properties, as GPx release correlates with the acute phase of inflammation [25].

While MAR shows moderate inhibitory activity against lipoxygenase (LOX), an enzyme involved in lipid mediator generation and immune response modulation [26], according to screening using the SwissTargetPrediction online tool [27], arachidonate 5-lipoxygenase was revealed as one of the potential targets for MAR (with a probability of 0.105); its potency (IC_50_ = 173 μg/mL) is superior to that of other *Marrubium* species extracts [28]. Compared with the standard drug used here, diclofenac with IC_50_ = 7.1 μg/mL, MAR displayed inferior potency. However, extracts from other Lamiaceae species are known to have significantly lower IC_50_ values; extracts obtained from two *Clerodendrum* species had IC_50_ values in the range of 14.1–30.7 μg/mL [29]. Additionally, MAR displays differential toxicity towards leukocytes, with PMNCs being the most sensitive, highlighting its potential therapeutic specificity [30,31].

Since the window during which MAR can affect inflammatory cell viability and function is approximately 5 h, an experiment was designed with a timed interval of 5 h. Each hour, the viability of granulocytes, PMNCs, and spleen lymphocytes exposed to different concentrations of MAR was assessed (Table 2). PMNCs, granulocytes, and spleen lymphocytes were found to be the most sensitive cells to the action of MAR, respectively. Glucocorticoids, steroid-based anti-inflammatory drugs, induce cytolysis in different populations of leukocytes at varying time points depending on cell activity and maturity [30], which partially overlaps with the observed activity of MAR. Conversely, no difference in response to glucocorticoids compared with lymphocytes was observed in neutrophils exposed to proinflammatory stimuli [31]. In this study, the toxicity of MAR towards different leukocytes may not be related to their response to inflammatory stimuli but to other features of these cells. Additionally, the concentrations of MAR tested in vitro could potentially correlate with the concentrations reached in tissue after systemic application of MAR, especially at concentrations of 10^−7^ and 10^−8^ M. At these concentrations, after 4 and 5 h of exposure, MAR caused a decrease in granulocytes and PMNCs, which are cells primarily associated with acute inflammation and whose decrease was observed (Figure 1 and Figure 2D).

## 4. Materials and Methods

### 4.1. General

All solvents (HPLC grade) were purchased from Sigma-Aldrich (St. Louis, MO, USA) and Fisher Chemical (Pittsburgh, PA, USA). Streptomycin, penicillin G, and 3-(4,5-dimethylthiazol-2-yl)-2,5-diphenyltetrazolium bromide (MTT) were purchased from AppliChem (Darmstadt, Germany). The cell medium (RPMI, acquired from Sigma-Aldrich, USA) used in the experiments consisted of RPMI 1640 with 20 mM HEPES and l-glutamine, without sodium bicarbonate, containing 10% (*v*/*v*) fetal bovine serum, 200 mg mL^−1^ streptomycin, and 200 IU mL^−1^ penicillin. Silica gel 60, particle size distribution 0.02–0.045 mm (Carl Roth, Karlsruhe, Germany), was used for column chromatography. Thin-layer chromatography (TLC) was performed on Merck plates (Darmstadt, Germany), layer thickness 0.2 mm with silica gel 60 and fluorescence indicator F_254_. Visualization was accomplished by spraying the plates with a mixture of sulfuric and nitric acids (1:1) followed by short, gentle heating. Melting point was determined on a Büchi (Flawil, Switzerland) M-560 melting point apparatus. Optical rotation was measured in chloroform on an Autopol IV (Rudolph Research Analytical, Flanders, NJ, USA) polarimeter equipped with a sodium lamp (589 nm) and a 1 mL cell with a 1 dm path length.

### 4.2. Plant Material

Above-ground parts of *Marrubium peregrinum* L. (Lamiaceae) were collected in October 2013 near Niš in southern Serbia (43°17′48″ N, 21°55′16″ E). The curator of the Herbarium confirmed the identity of the material.

### 4.3. Isolation of MAR

Homogenized dry plant material (600 g) was extracted by maceration at room temperature with a mixture of 1 L of hexane, 2 L of diethyl ether, 1.5 L of ethyl acetate, and 0.5 L of methanol for 7 days, with occasional shaking. Following filtration, the solvent was removed using a rotary evaporator and 15.5 g of extract was obtained. The extract (15.0 g) was initially separated by dry-flash chromatography on silica gel (gradient elution, from pure hexane to pure diethyl ether). Fractions 17–19 (1.29 g, eluted with Et_2_O–hexane 3:1, *v*/*v*) were pooled and rechromatographed on a Sephadex LH-20 column (Merck, Germany, MeOH-CHCl_3_ 1:1, *v*/*v*) and 0.79 g of MAR was isolated. The isolated compound was deemed pure via TLC and ^1^H NMR and was not purified further. NMR data correlate well with those reported by Yamakoshi et al. [10]. *R*_f_ = 0.24 (Et_2_O–hexane 1:1), m.p. 157–158 °C (lit. 160–161 °C [10]), [α]_D_^20^ +36.0 (*c* 1.2, CHCl_3_) (lit. [α]_D_^20^ +34.4 (*c* 1.04, CHCl_3_) [10]).

### 4.4. NMR Spectroscopy

The ^1^H and ^13^C NMR spectra of MAR were recorded on a Bruker Avance III spectrometer (Bruker, Fallanden, Switzerland), operating at 400 and 100.6 MHz, respectively. The 2D experiments (NOESY and gradient ^1^H–^1^H COSY, HSQC, HMBC), as well as DEPT-90, DEPT-135, and selective ^1^H homonuclear decoupling measurements, were run on the same instrument with the built-in Bruker pulse sequences. NMR spectra were measured at 25 °C in CDCl_3_ with chemical shifts δ (in ppm) referenced to internal tetramethylsilane.

### 4.5. Animals and Housing

In this study, C57BL/6 mice weighing 25–30 g were used. The animals were kept in IVC cages, with a 12/12 h light/dark cycle, free access to water and food, and controlled room temperature (21 ± 1 °C) in the vivarium of the Institute for Biomedical Research, Faculty of Medicine in Niš. All experimental procedures with the animals were conducted in accordance with the Declaration of Helsinki and European Community guidelines for the ethical handling of laboratory animals (EU Directive of 2010; 2010/63/EU), as suggested by the National Law of Animal Welfare (the *Official Gazette of the Republic of Serbia,* No.: 41/2009 and 39/2010). These procedures were also approved by the Ethics Committee (No. 12-2466-6).

### 4.6. In Vivo Anti-Inflammatory Activity Determination

#### 4.6.1. Carrageenan-Induced Peritonitis Induction

On the day of the experiment, animals were randomly assigned to seven groups (*n* = 7) and treated as follows:

- Control group—vehicle (olive oil) at a dose of 10 mL/kg
- Positive control group—indometacin at a dose of 10 mg/kg
- Positive control group—diclofenac at a dose of 30 mg/kg
- Experimental group—MAR at a dose of 1 mg/kg
- Experimental group—MAR at a dose of 10 mg/kg
- Experimental group—MAR at a dose of 20 mg/kg
- Experimental group—MAR at a dose of 40 mg/kg

One hour after the treatment, the animals were intraperitoneally injected with 1% (*w*/*w*) carrageenan in a volume of 250 μL [18]. Four hours after the treatment, the animals were euthanized with 10% (*w*/*w*) ketamine (Richter Pharma, Wels, Austria) and afterward injected with warm PBS. The abdomen was gently massaged, and its contents were aspirated and kept on ice until centrifugation. Peritoneal lavages were centrifuged at 1200 rpm for 10 min at 4 °C, the supernatant was removed, and the peritoneal exudate cell (PEC) pellet was resuspended in 1 mL of PBS. The separated supernatants were frozen at −80 °C for biochemical analysis, while the resuspended cells were used for further analysis.

#### 4.6.2. Peritoneal Exudate Cell Count and Differential Staining

The total number of PECs was counted in a Neubauer counting chamber after appropriate staining with trypan blue [32]. After staining, the cell density for further cultivation was adjusted to 2.5 × 10^6^ cells/mL, and the cells were further placed in sterile 96-well plates and left in an incubator at 37 °C. After a 1.5 h incubation, the cells were utilized for various functional tests.

In parallel, smears containing PECs, prepared from the cell suspension obtained through peritoneal lavage, were stained with May–Grünwald Giemsa for a differential cell count [33]. The slides were immersed in a May–Grünwald solution, rinsed with distilled water, and stained with Giemsa. After staining, the slides were washed, air-dried at room temperature, and examined under an immersion lens (×1000) using a light microscope (Carl Zeiss, Gottingen, Germany). The percentage of mononuclear (agranulocytes) or polymorphonuclear cells (granulocytes) was calculated based on 200 observed PECs.

#### 4.6.3. Scanning Electron Microscopy of PECs

For scanning electron microscopy (SEM), cover plates with adhered cells obtained from the pellet after centrifugation were fixed with a 2.5% glutaraldehyde solution diluted in PBS. They were then rinsed with PBS, postfixed with 1% OsO_4_ solution for 1 h, rinsed again, dehydrated in graded alcohol solutions (ranging from 30% to 100%) and dried afterward. Gold coating was applied using a sputter coater, and the cells were examined using a JEOL JSM 5300 (JEOL Ltd., Tokyo, Japan).

#### 4.6.4. Biochemical Analysis of the Peritoneal Exudate Fluid

Peritoneal fluid with cells was centrifuged at 10,000 rpm for 10 min at 40 °C. Subsequently, the protein content was determined via Lowry’s method, with bovine serum albumin as the quantification standard. The activity of free MPO was measured through the amount of oxidized *o*-phenylenediamine in a reaction that included peritoneal exudate fluids (supernatants) and H_2_O_2_. The absorbance of the reaction product was recorded at 540 nm [33]. The previously described method determined the reduced glutathione (GSH) content in supernatants [34]. The method is based on the reaction between non-protein thiols and DTNB (5,5′-dithiobis-(2-nitrobenzoic acid)) reagent at room temperature. The final amount of GSH in peritoneal fluid, expressed as nmol/mg of proteins, was calculated based on the standard curve constructed using GSH. Glutathione peroxidase (GPx) activity in samples was determined based on the extent of the reaction between H_2_O_2_ and exogenously added GSH [35]. The final GSH content was determined as described previously, and the enzyme activity was expressed as nmol/min/mg of proteins.

#### 4.6.5. Mesenterial Tissue Staining for Mastocyte Degranulation

At the end of the experiment, from each animal, a piece of the mesentery tissue was isolated and fixated/stained for 30 min using 10% formalin containing toluidine blue (0.1%) and acetic acid (1%) [36]. The stained mesentery tissue was then mounted on a glass slide, taking care not to fold or stretch the tissue sample. Mast cell degranulation was observed under a light microscope at a magnification of 200×. At least 100 mast cells were counted per sample, from which the percentage of degranulation was calculated. Non-degranulated mast cells were easily identified, appearing dark blue with condensed granules covering the entire cell surface. In contrast, degranulated mast cells were characterized by extruded granules around the surface and in the vicinity. The data are expressed as a percentage of degranulation compared with the values in the control group.

### 4.7. In Vitro Effects of MAR on Inflammatory Cell Viability—Time Lap Study

Healthy C57 mice were used to isolate peripheral mononuclear cells (PMNCs) and granulocytes from blood, while spleen tissue was used to isolate a mixed population of T and B lymphocytes [37]. PMNCs and granulocytes were isolated from blood samples obtained by cardiac puncture using vacutainer tubes (3 mL volume) coated with EDTA. After centrifugation on a Ficoll^®^ (Sigma-Aldrich, St. Louis, MO, USA) gradient, the buffy coat was used as a source of PMNCs, while the bottom layer containing mainly red blood cells was lysed (using ammonium chloride) and washed several times to obtain a cell pallet containing only granulocytes. Both cell samples were stained with trypan blue, and the cell density was adjusted to 10^6^/mL of RPMI. After the blood was withdrawn, mouse spleens were aseptically removed, finely chopped into smaller pieces, and strained through a 20 μm mesh strainer using PBS with the addition of ammonium chloride. Isolated splenocytes were stained with trypan blue to determine their viability, and the cell density was adjusted to 10^6^/mL of RPMI. The final concentration of cells for plating and short-term cultivation in 96-well plates was 10^6^/mL of RPMI. Cells were further incubated at 37 °C under 5% CO_2_ for up to 5 h in the presence of MAR.

Cells were treated with different concentrations of MAR ranging from 10^−4^ to 10^−8^ M to analyze potential in vitro effects on their viability. The negative control group comprised cells that were incubated only with medium (RPMI) without added substances. After adding the test substance, plates were returned to incubation, and cell viability was monitored after 1, 2, 3, 4, and 5 h of incubation, attempting to mimic the 5 h period of the in vivo experiment. After each hour, the cell medium was removed, fresh medium containing MTT (1 mg/mL) was added to all wells, and the previously described procedure was repeated.

### 4.8. Lipoxygenase Inhibition—In Vitro Study

The lipoxygenase (LOX) inhibition activity of MAR was assessed through a modified procedure derived from Gunathilake et al. [38]. MAR (0.05 M) stock solution was prepared by dissolving 166.2 mg of MAR in 10 mL of methanol. Sodium linoleate (0.01 M) solution was prepared by following a published procedure [39]: 70 mg of linoleic acid and 70 mg of Tween 20 were homogenized in 4 mL of water, 0.5 mL of 0.5 M NaOH was added, and the obtained clear solution was diluted to 25 mL. 

Lipoxygenase (8000 U/mL, 100 μL) and MAR (final concentration: 0.0001–0.001 M, 1 mL of solution obtained by diluting the stock solution with borate buffer, pH 8.8, 0.1 M) were mixed with 2 mL of sodium borate buffer (pH 8.8, 0.1 M) and the obtained solution was incubated for 5 min. Sodium linoleate (20 μL) solution was added, and the absorbance of the mixture was measured at 234 nm over time (UV-1800 spectrophotometer, Shimadzu, Tokyo, Japan). The same procedure was used to determine the LOX inhibition activity of the positive control (diclofenac, in concentration range 2.5–25 μg/mL).

### 4.9. Statistical Analysis

Data presented as mean ± SD were compared using one-way analysis of variance (ANOVA), followed by Tukey’s post hoc test for multiple comparisons (GraphPad Prism, Boston, MA, USA). Probability values (*p*) less than 0.05 were considered to be statistically significant.

## 5. Conclusions

Crude extracts typically comprise numerous constituents that may synergistically contribute to antioxidant and/or cytotoxic activities. Therefore, the observed activity of the extract may not be directly attributable to the activity of MAR observed here. Marrubiin demonstrated the inhibition of peritoneal inflammation induced by carrageenan, preventing inflammatory cell infiltration and peritoneal mast cell degranulation. The activity was further substantiated through a series of biochemical parameters, revealing that the peritoneal fluid of animals treated with MAR exhibited lower protein levels and reduced MPO activity compared with the untreated animals. Additionally, in vitro experiments conducted over 5 h demonstrated MAR’s pronounced cytotoxic activity towards isolated PMNCs, the primary contributors to peritoneal inflammation. However, further studies with MAR are needed to elucidate detailed molecular mechanisms, providing a comprehensive understanding of the activity of this potent anti-inflammatory molecule.

## Figures and Tables

**Table 1:** Effects of MAR on inflammatory parameters in peritoneal exudate fluid.

Dose/Parameter	Proteins (μg/mL)	MPO (OD × 1000)	GSH (nmol/mg of Proteins)	GPx (nmol/min/mg of Proteins)
Vehicle 10 mL/kg	77 ± 5	128 ± 14	8.4 ± 4.1	4.0 ± 0.1
Diclofenac 5 mg/kg	45 ± 10 *	76 ± 14 *	13 ± 3	6.6 ± 1.2 *
Indometacin 10 mg/kg	40 ± 7 *	84 ± 13 *	6.8 ± 2.2	2.6 ± 0.4 *
MAR 1 mg/kg	55 ± 17 **	81 ± 16 *	13 ± 6	6.1 ± 0.4 *
MAR 10 mg/kg	58 ± 4 **	86 ± 17 **	12 ± 5	6.8 ± 0.2 *
MAR 20 mg/kg	55 ± 2 *	90 ± 15 **	9.7 ± 2.8	5.2 ± 0.3 **
MAR 40 mg/kg	47 ± 4 *	84 ± 7 *	13 ± 2	5.5 ± 0.5 *

Data are given as mean ± SD and were compared using one-way ANOVA followed by Tukey’s post hoc test, * *p* < 0.01, ** *p* < 0.001 vs. vehicle-treated animals.

**Table 2:** Effect of MAR on different inflammatory-cell viability estimated by MTT reduction assay during a time lapse study.

Time (h)/ Concentration (M)	MAR				
	10^−4^	10^−5^	10^−6^	10^−7^	10^−8^
Granulocyte viability compared with the control (%)					
1	101 ± 6	120 ± 10	113 ± 11	118 ± 9	107 ± 6
2	82 ± 8 **	106 ± 4	108 ± 6	98 ± 3	97 ± 3
3	96 ± 5	91 ± 5	101 ± 7	98 ± 6	94 ± 6
4	80 ± 9 **	96 ± 6	92 ± 9	111 ± 12	98 ± 6
5	69 ± 5 *	72 ± 12 *	70 ± 14 *	78 ± 9 *	79 ± 10 *
PMNCs viability compared with the control (%)					
1	115 ± 9	97 ± 5	109 ± 6	112 ± 8	103 ± 7
2	105 ± 8	112 ± 8	115 ± 9	100 ± 5	99 ± 2
3	63 ± 11 *	59 ± 12 *	67 ± 17 *	75 ± 12 *	93 ± 11
4	54 ± 16 *	55 ± 11 *	62 ± 12 *	70 ± 4 *	88 ± 5 **
5	46 ± 10 *	50 ± 13 *	53 ± 11 *	61 ± 7 *	80 ± 2 **
Spleen lymphocyte viability compared with the control (%)					
1	96 ± 5	95 ± 4	102 ± 7	108 ± 66	102 ± 5
2	102 ± 4	95 ± 7	106 ± 5	97 ± 6	96 ± 4
3	98 ± 4	93 ± 5	94 ± 4	87 ± 10	105 ± 2
4	90 ± 2 **	102 ± 8	98 ± 4	114 ± 9	101 ± 4
5	79 ± 7 *	92 ± 7	103 ± 4	102 ± 5	95 ± 3

* *p* < 0.001, ** *p* < 0.01 vs. RPMI-treated cells.

## Data Availability

Data are contained within the article and Supplementary Materials.

## Supplementary Materials

The following supporting information can be downloaded at: https://www.mdpi.com/article/10.3390/ijms25084496/s1.
