# Supplementary material for: Marrubiin Inhibits Peritoneal Inflammatory Response Induced by Carrageenan Application in C57 Mice
Source: Int J Mol Sci. 2024 Apr 19;25(8):4496. doi: 10.3390/ijms25084496 (PMC11050121; doi:10.3390/ijms25084496)
Supplement: Supplementary file 1 [file ijms-25-04496-s001.zip › ijms-2969186-supplementary.pdf]

Supplementary Material

Marrubiin Inhibits Peritoneal Inflammatory Response Induced by Carrageenan Application in C57 Mice

Niko S. Radulović [1,*], Miljana R. Đorđević Zlatković [1], Nikola M. Stojanović [2], Milan S. Nešić [1], Dragan B. Zlatković [1], Milena S. Potić Floranović [3], Dragana S. Tričković Vukić [3] and Pavle J. Randjelovic [2]

[1] Department of Chemistry, Faculty of Sciences and Mathematics, University of Niš, 18000 Niš, Serbia; miljana.djordjevic@pmf.edu.rs (M.R.Đ.Z.); nesicmilanvl@gmail.com (M.S.N.); dragan.zlatkovic@gmail.com (D.B.Z.)

[2] Department of Physiology, Faculty of Medicine, University of Niš, 18000 Niš, Serbia; nikola.st90@yahoo.com (N.M.S.); pavleus@gmail.com (P.J.R.)

[3] Scientific Research Centre for Biomedicine, Faculty of Medicine, University of Niš, 18000 Niš, Serbia; milenapotic@yahoo.com (M.S.P.F.); draganavukic18@gmail.com (D.S.T.V.)

* Correspondence: nikoradulovic@yahoo.com

Table of contents: page

Figure S1: ^{1}H NMR spectrum of marrubiin (MAR) 2
Figure S2: ^{13}C NMR spectrum of MAR 2
Figure S3: ^{1}H NMR spectrum of MAR after 10 years of storage at 4 ◦C 3

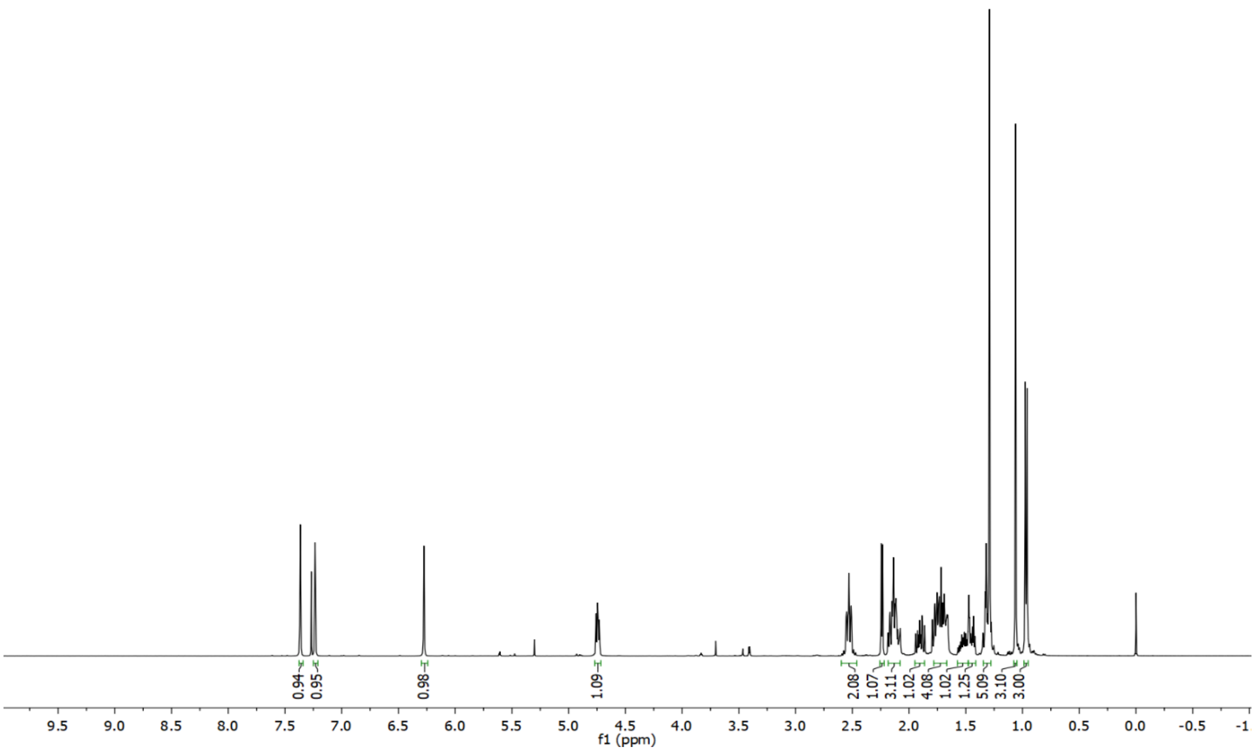

Figure S1. ^{1}H NMR (400 MHz) spectrum of marrubiin (MAR) in $CDCl_3$

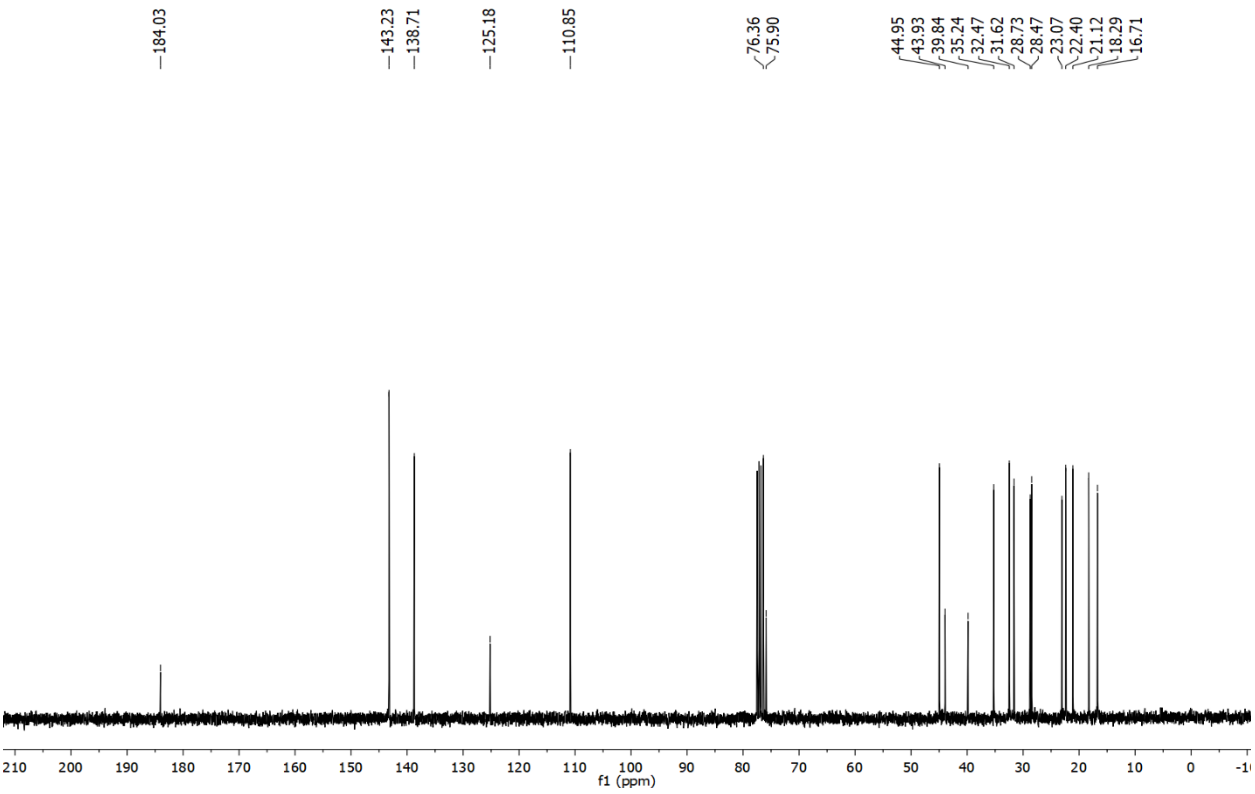

Figure S2. ^{13}C NMR (100.6 MHz) spectrum of MAR in $CDCl_3$

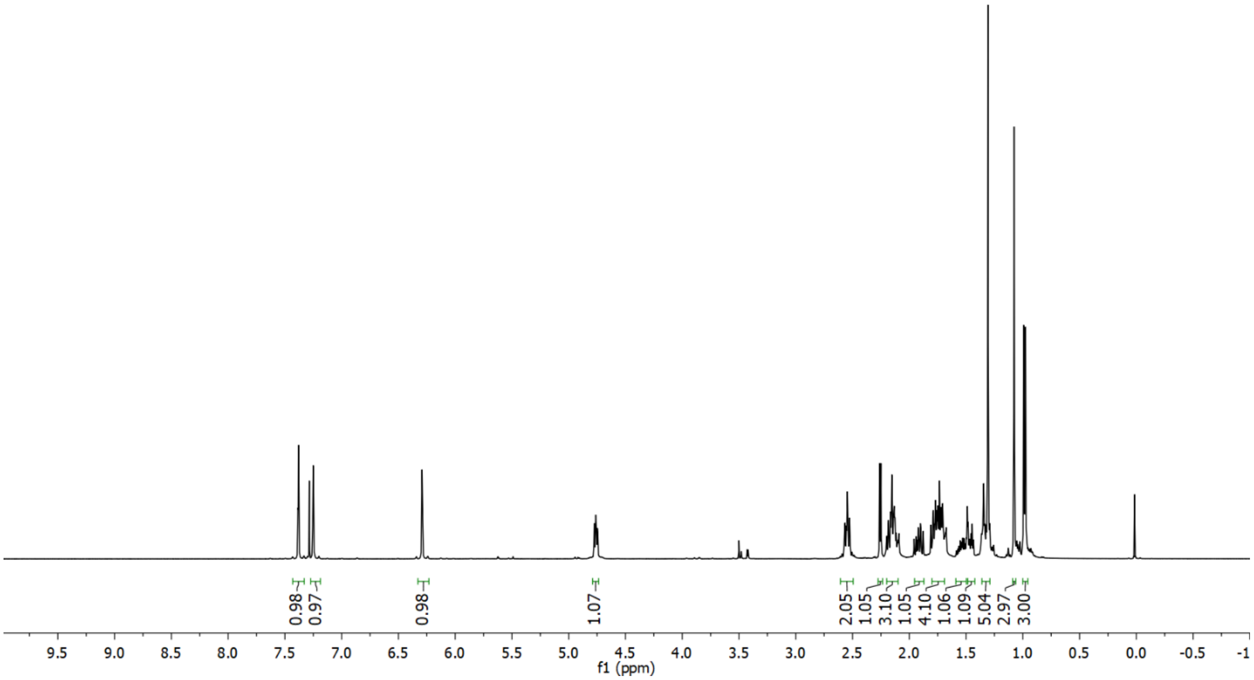

Figure S3. ^1H NMR (400 MHz, $CDCl_3$) spectrum of MAR after 10 years of storage at 4 ◦C. No degradation of MAR can be observed.
